# Proof of Concept: Measuring Aortic Annulus Resistance by Means of Pressure-Volume Curves During Balloon Inflation to Guide Transcatheter Aortic Valve Implantation

**DOI:** 10.3389/fcvm.2021.665029

**Published:** 2021-04-30

**Authors:** Timothée Noterdaeme, Jonas Gesenhues, Felix Vogt, Steffen Massberg, Nikolaus Marx

**Affiliations:** ^1^Department of Cardiology & Intensive Care Medicine, University Hospital Aachen, RWTH Aachen University, Aachen, Germany; ^2^Institute of Automatic Control, RWTH Aachen University, Aachen, Germany; ^3^Munich University Center, Ludwig-Maximilians University, and German Centre for Cardiovascular Research (DZHK), Partner Site Munich Heart Alliance, Munich, Germany

**Keywords:** aortic valve stenosis, transcatheter aortic valve implantation, pressure volume curve, atrioventicular block, paravalvular leak, translational research

## Abstract

This study assessed the basic working principle to measure aortic annulus resistance during balloon inflation for transcatheter aortic valve implantation (TAVI), by acquisition of pressure-volume curve for a guided semi-automatic implantation. A modular bench-system was used which allows the incremental inflation of valvuloplasty balloons by means of a stepper-motor driven linear axis with simultaneous recording of the pressure changes inside the system. Different porcine aortic xenografts were assessed by use of a non-compliant valvuloplasty balloon. In a second step transcatheter aortic stents were implanted inside target sized xenografts. The recorded pressure volume-curves showed that the system can accurately differentiate between different xenografts and assess the quality of the tissue rendering real-time analysis of pressure-volume curves during balloon-inflation possible, which has the potential to optimize the implantation procedure by direct adaptation to the patient specific anatomy and characteristics. Further investigations and development are warranted.

## Introduction

Recently, transcatheter aortic valve implantation (TAVI) for aortic stenosis has moved toward the treatment of low-risk patients with good short-term results (1). In this younger population, the lingering problems of paravalvular leakage (PVL), valve deterioration as well as the still existing necessity for pacemaker implantation, even with the latest TAVI-generation (2, 3), might affect the long-term outcome and hinder further implementation. Several studies have shown, that a patient-specific device-host interaction determines valve geometry (4, 5) therefore having a direct effect on the incidence and severity of the above-mentioned complications. Furthermore, the anchoring principles and radial force exerted by the frame varies greatly between different TAVI prostheses. Whereas, valves with a nitinol frame actively exert a radial force when compressed due to their super-elastic properties, valve stents made from chrome-cobalt alloy only passively oppose radial compression with no active force component. The interaction between the host and the used TAVI prostheses can be approximated beforehand by computer-based simulations yet remains fragmented due to the high variability of the individual tissue characteristics of each patient. Therefore, this information has remained almost impossible to implement during the implantation procedure itself. Current implantation techniques do not take this important point into consideration, though the quantification of this interaction during the implantation with an individualized adaptation to local tissue characteristics has tremendous potential for improving TAV implantation and might be crucial for achieving better long-term results.

In the present study, we evaluated the concept of acquiring pressure-volume curves during balloon inflation to directly assess local tissue characteristics of the aortic annulus, which could potentially be used to control a semi-automated implantation system for guiding the expansion and final modulation of TAVI prostheses inside the native aortic valve.

## Materials and Methods

**Figure 1:**
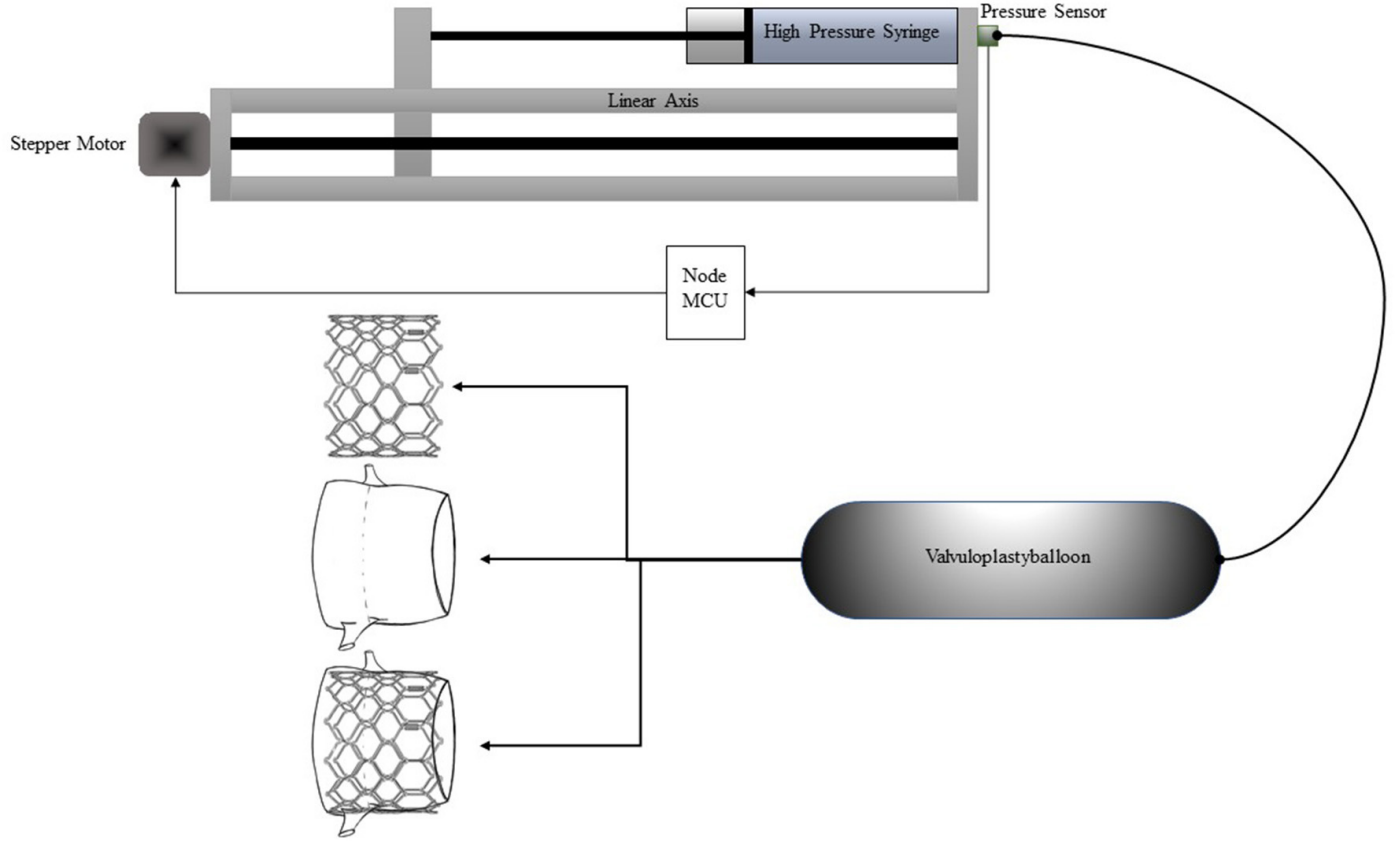
Schematic representation of the setup.

A modular benchtop system (Figure 1) was devised in which a stepper motor driven (Nema 34) linear axis incrementally inflates a non-compliant valvuloplasty balloon (Bard True Dilatation 24 mm) with water while simultaneously recording the pressure variation through a connected pressure sensor (Seeed technology: SKU114991178). The amplitude of movement of the linear axis was chosen so that the applied volume generates an internal pressure of 4 bars inside the system, corresponding to 1 bar above the nominal pressure of the used valvuloplasty balloon. Data-points were acquired every 10 ms via a NodeMCU board as well as for every micro-step of the stepper-motor and recorded as CSV-file. Graphics were plotted with Microsoft Excel. After calibration, the valvuloplasty-balloon was inflated inside different undersized porcine aortic xenografts (11, 13, and 15 mm) purposely leading to their rupture. The size of the xenografts annulus was measured beforehand using standard surgical sizing instruments. In a second setup, the system first recorded the pressure profile during dilatation of a generic chrome-cobalt frame (24 mm outside diameter) crimped directly onto the valvuloplasty balloon. Afterwards, an identical frame was dilated inside a target sized porcine xenograft (22 mm) to simulate the implantation and assess the pressure curve during the procedure. All curves are plotted as function of the absolute position of the stepper motor, expressed in steps corresponding directly to the applied volume.

## Results

### Xenograft Assessment

**Figure 2:**
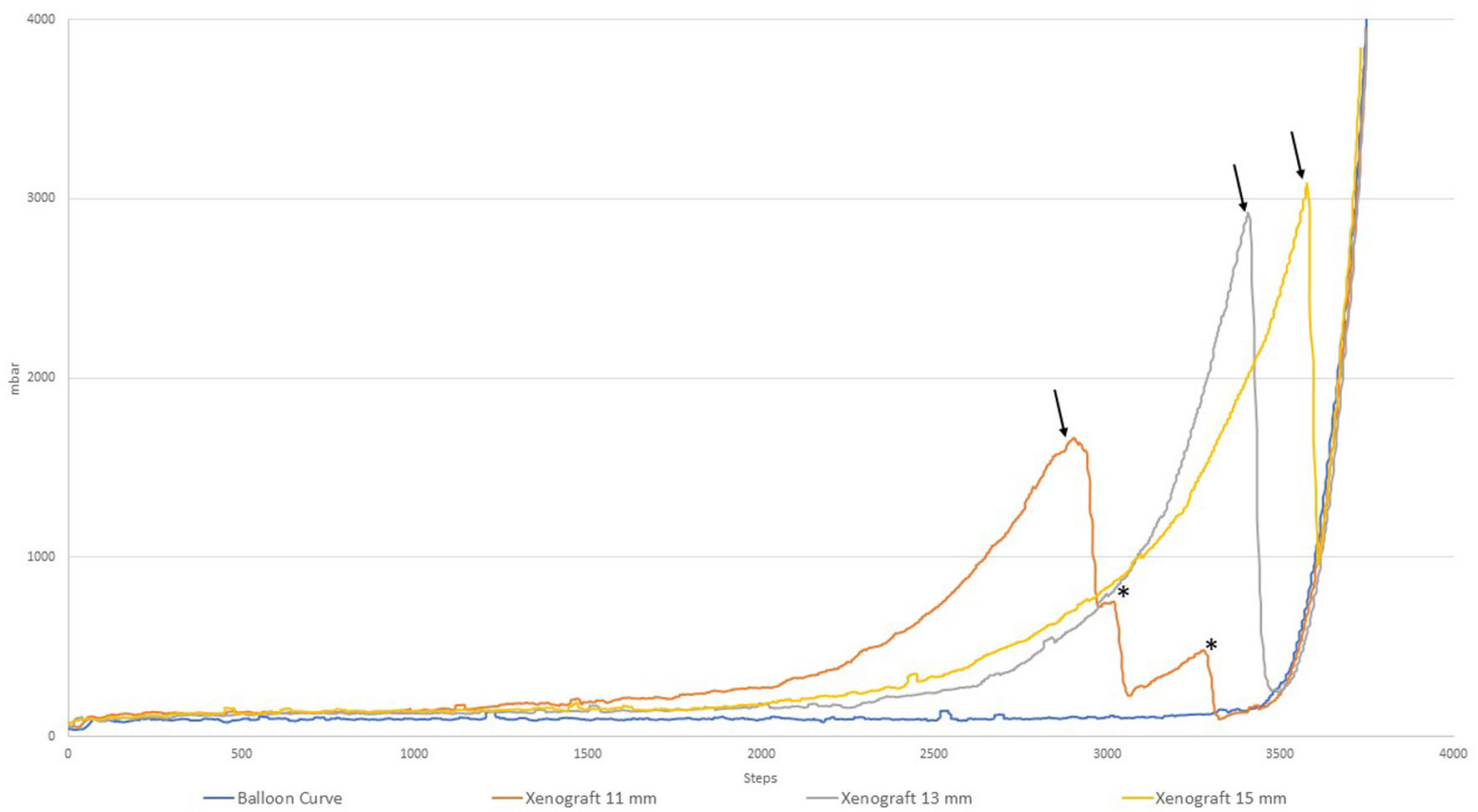
Pressure-volume curves of different xenografts.

Pressure-volume curves of the different sized xenografts are shown in Figure 2 in comparison to the curve of the balloon. For the different xenografts (11, 13, and 15 mm), the point when the internal pressure begins to rise, the inclination itself, the rate in which the inclination increases as well as the respective peaks are clearly detectable and can be used as parameters to characterize and distinguish the xenograft from each other. The values of the different xenograft are summarized in Supplementary Table 1.

The point at which the pressure starts to increase (defined as a pressure > 200 mbar or 150 mmHg) corresponds to the moment when the non-compliant balloon has established a firm contact with the xenograft and is capable to exert force on it. For the 15 mm Xenograft this point is reached earlier than in the 13 mm one which can be explained by either another part of the xenograft, for example, the outflow-tract, having a smaller diameter than the one measured at the annulus or that the manual measuring accuracy is imperfect as the tissue is evidently capable of stretching. The inclination expressed in dP/dV corresponds to the stiffness of the xenograft, becoming gradually stiffer toward the end. This increase could, in turn, be expressed in d^2^P/dV^2^, or the second derivation of the acquired curve. The occurring ruptures can be detected by a sharp decrease in the corresponding curve (marked by an arrow). Of note are the additional peaks that can be seen in the 11 mm xenograft corresponding to first the rupture of the annulus and afterwards wider parts of the ascending aorta (marked by an asterisk).

### Stent and Implantation Assessment

**Figure 3:**
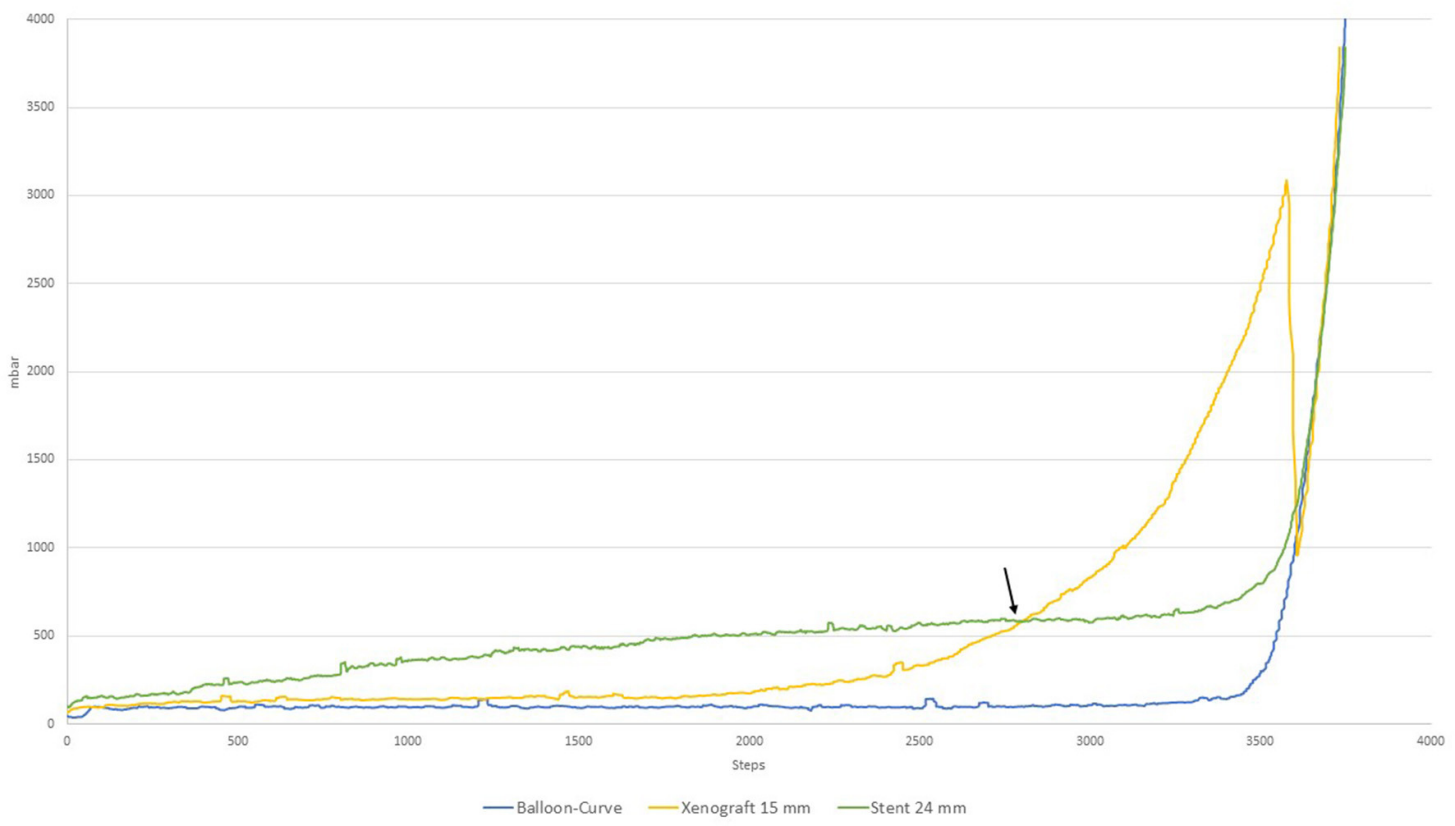
Radial resistance of 24 mm chrome-cobalt TAVI frame vs. 15 mm Xenograft.

In Figure 3 the pressure-volume curves of the generic 24 mm chrome-cobalt frame stent and the 15 mm Xenograft are shown. Of particular interest is the intersection of both curves at which the radial resistance of the stent equals the exerted force by the tissue. Before the point of intersection (marked by an arrow) the push-back by the anatomy would not be sufficient for adequate anchoring. After this point, the stent would be submitted to a higher outside force than its own radial resistance leading to a tissue-specific amount of recoil of the stent depending on the remaining elasticity of the xenograft. With further inflation the potential effective orifice area of the stent can be increase at the detriment of approaching the point of annular rupture marked by the sharp drop in the curve of the 15 mm xenograft. Similarly, the amount of paravalvular leakage as well as the risk of inducing permanent conduction disturbances are at opposite sides of the spectrum. The optimal amount of inflation is located between those two points with, however a narrow margin of 5.2 ml (780 steps) between anchoring and aortic rupture.

**Figure 4:**
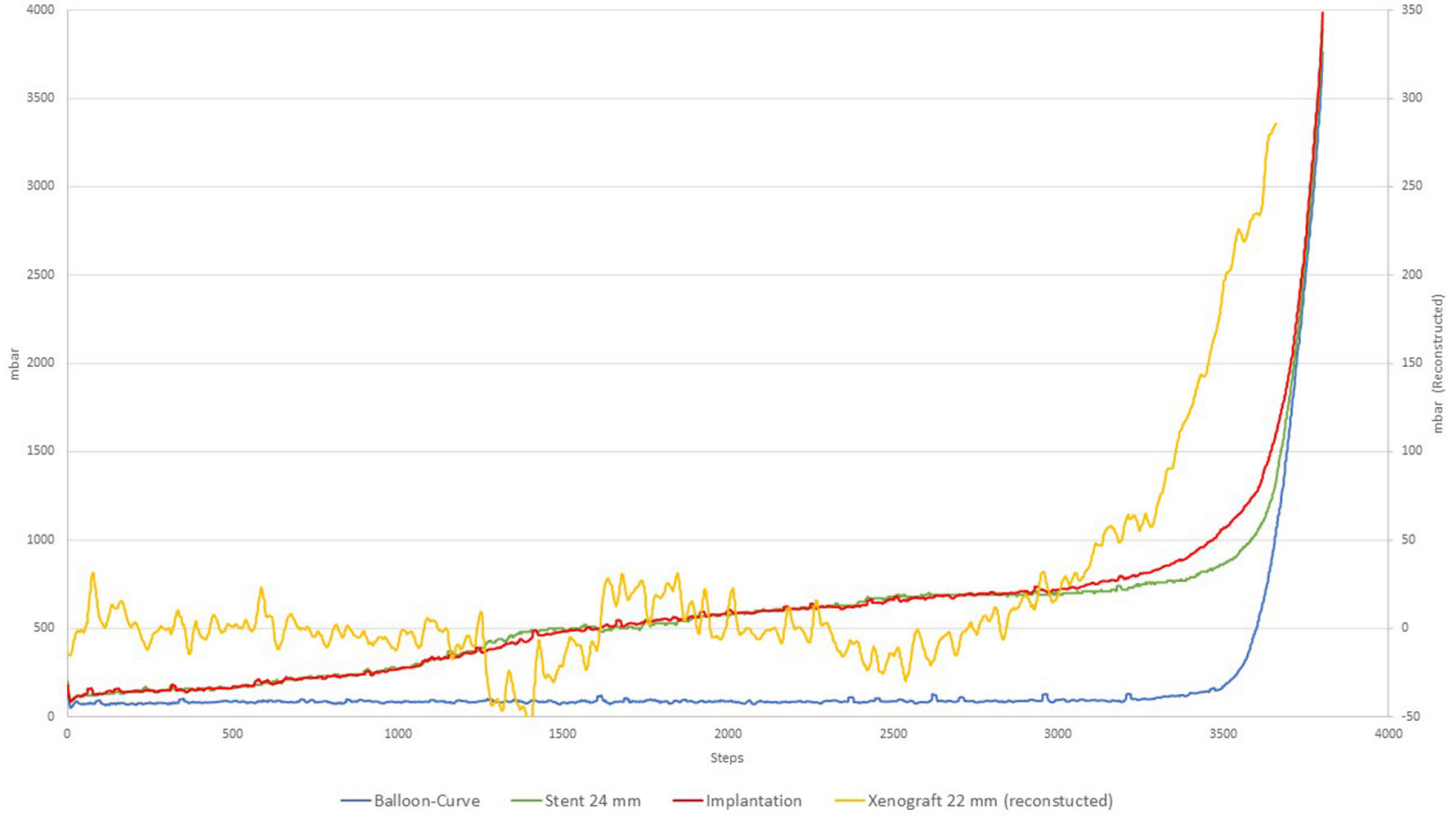
Implantation procedure.

The implantation procedure is depicted in Figure 4 in which a 22 mm porcine xenograft was used. The characteristics of the xenograft can be reconstructed by subtracting the curves from each other and used for analysis similar as above. The resulting curve is depicted in yellow and shows the initial rise of the tissue resistance similar to those observed in Figure 1.

## Discussion

Our study demonstrates that pressure-volume curves and their real-time analysis can be used to guide the implantation of TAVI-protheses to match the patient's specific anatomy. Such a patient-tailored approach may help to address the existing shortcomings of current valve implantation techniques such as paravalvular regurgitation or conduction disturbances.

Manually inflating any type of valvuloplasty-balloon, solely relying on exerted pressure, or applied volume remains a “rough” approach during TAVI. Inflating to a specified pressure does not guarantee full expansion to the nominal diameter while inflating to a specified volume does not ensure that the required pressure has been achieved or even worse has been exceeded. The registration of pressure-volume curves during implantation however allows to quantify the compliance of a stenotic valve as well as the surrounding tissue during the implantation process. The analysis of the pressure-volume curve would assure that the frame is sufficiently inflated, compensating for recoil and ensures that the exerted pressure does not exceed a certain predefined value to avoid complications such as conduction disturbances or annular rupture, especially as the margin between adequate anchoring and rupture can be narrow. Furthermore, if during the analysis it is apparent that the compliance would allow for a slightly bigger diameter, inflation could be continued to increase the effective orifice area of the prostheses. As the relation between diameter and effective orifice area is exponential, even a minute increase can be sufficient to decrease the transvalvular gradient even further. Preprocedural errors in determining the correct aortic annulus diameters can be compensated with these measurements, as this technique not only reflects the aortic annulus itself but also take into consideration other variants that are difficult to predict or determine in advance.

Before human application, several technical limitations need to be addressed of which the current slow inflation speed of the system is one. Different solutions to this problem exist of which the use of non-linear inflation curves represents several advantages. As ¾ of the inflated volume are necessary to expand the stent from its initial crimped form before it comes into contact with the aortic annulus, it could be applied with higher inflation-speed and lower pressure resolution or even be completely ignored. Once the stent approaches the aortic structure the speed of volume-application can be reduced as to enhance the resolution in pressure differential and to modulate the implantation to the encountered tissue characteristics. As the state of expansion of the stent is always known from the applied volume, calcification of the leaflets could be taken out of the equation as the potential pressure increases would occur in the first part of the inflation process before the minimum target volume has been achieved. Further thorough investigations with *in vitro* studies as well as a comparative *in-vivo* animal study need to be conducted to investigate the proposed solutions before finding their way into the Cath-lab. It is however important to note that even animal studies remain a rough approximation for aortic stenosis due to the lack of calcification and might be of limited significance. However, it would be possible to use the system initially to standardize the existing implantation procedure and help record the encountered variabilities in a first step and correlate these findings with the resulting outcome. This would provide valuable information for a complete future automation.

As the setup is comprised of only a linear axis and a pressure sensor, with very limited space requirements, it can be easily adapted to already existing solutions and materials with minimum changes in the workflow and be used in a broad band of application. Pre-dilatation of the native aortic valve to match the radial force of the used frame or post-dilatation to reduce remaining PVL are also key aspects for which the system could be used. Furthermore, the system might resolve the conundrum of TAVI in patients with pure aortic regurgitation without calcification. Matching the radial resistance of the prostheses to the compliance of the aortic annulus could assure sufficient anchoring. Careful pre-operative planning to determine controlling parameters guiding the system such as minimum and maximum inflation volume, as well as a slightly different approach to oversizing and implantation might be necessary for the system to perform adequately.

## Data Availability Statement

The raw data supporting the conclusions of this article will be made available by the authors, without undue reservation.

## Author Contributions

TN, JG, and NM contributed to conception and design of the study. TN wrote the first draft of the manuscript. TN, JG, SM, and NM wrote sections of the manuscript. SM and NM provided insides into the subject. All authors contributed to manuscript revision, read, and approved the submitted version.

## Conflict of Interest

The authors declare that the research was conducted in the absence of any commercial or financial relationships that could be construed as a potential conflict of interest.
