# Supplementary material for: Proof of Concept: Measuring Aortic Annulus Resistance by Means of Pressure-Volume Curves During Balloon Inflation to Guide Transcatheter Aortic Valve Implantation
Source: Front Cardiovasc Med. 2021 Apr 30;8:665029. doi: 10.3389/fcvm.2021.665029 (PMC8119645; doi:10.3389/fcvm.2021.665029)
Supplement: Supplementary file 1 [file Table_1.DOCX]

Supplementary Material

# Supplementary Table

|  | Xenograft 11 mm | Xenograft 13 mm | Xenograft 15 mm |
| --- | --- | --- | --- |
| **X-value pressure increase** | 1451 (9,67 ml) | 2332 (15,54 ml) | 2144 (14,29 ml) |
| **Rupture Pressure** | 1665 mbar | 2922 mbar | 3086 mbar |
| **X-value rupture pressure** | 2905 (19,36 ml) | 3406(22,70 ml) | 3590 (23,93 ml) |
| **Average dP/dV** | 151,1 mbar/ml | 380,2 mbar/ml | 299,4 mbar/ml |
| **Peak dP/dV** | 544 mbar/ml | 1211 mbar/ml | 1190 mbar/ml |

**Supplementary Table 1:** Values of the different xenografts
